# Trends in catastrophic health expenditure in India: 1993 to 2014

**DOI:** 10.2471/BLT.17.191759

**Published:** 2017-11-30

**Authors:** Anamika Pandey, George B Ploubidis, Lynda Clarke, Lalit Dandona

**Affiliations:** a Public Health Foundation of India, Plot 47, Sector 44, Institutional Area, Gurugram 122 002, National Capital Region, India.; b Centre for Longitudinal Studies, UCL Institute of Education, London, England.; c Department of Population Health, London School of Hygiene & Tropical Medicine, London, England.

## Abstract

**Objective:**

To investigate trends in out-of-pocket health-care payments and catastrophic health expenditure in India by household age composition.

**Methods:**

We obtained data from four national consumer expenditure surveys and three health-care utilization surveys conducted between 1993 and 2014. Households were divided into five groups by age composition. We defined catastrophic health expenditure as out-of-pocket payments equalling or exceeding 10% of household expenditure. Factors associated with catastrophic expenditure were identified by multivariable analysis.

**Findings:**

Overall, the proportion of catastrophic health expenditure increased 1.47-fold between the 1993–1994 expenditure survey (12.4%) and the 2011–2012 expenditure survey (18.2%) and 2.24-fold between the 1995–1996 utilization survey (11.1%) and the 2014 utilization survey (24.9%). The proportion increased more in the poorest than the richest quintile: 3.00-fold versus 1.74-fold, respectively, across the utilization surveys. Catastrophic expenditure was commonest among households comprising only people aged 60 years or older: the adjusted odds ratio (aOR) was 3.26 (95% confidence interval, CI: 2.76–3.84) compared with households with no older people or children younger than 5 years. The risk was also increased among households with both older people and children (aOR: 2.58; 95% CI: 2.31–2.89), with a female head (aOR: 1.32; 95% CI: 1.19–1.47) and with a rural location (aOR: 1.27; 95% CI: 1.20–1.35).

**Conclusion:**

The proportion of households experiencing catastrophic health expenditure in India increased over the past two decades. Such expenditure was highest among households with older people. Financial protection mechanisms are needed for population groups at risk for catastrophic health expenditure.

## Résumé

**Objectif:**

Étudier les tendances en matière de paiement direct des frais de santé et de dépenses de santé catastrophiques en Inde selon la répartition par âge des ménages.

**Méthodes:**

Nous avons utilisé les données de quatre enquêtes nationales sur les dépenses des consommateurs et de trois enquêtes sur le recours aux soins, menées entre 1993 et 2014. Les ménages ont été divisés en cinq groupes suivant leur répartition par âge. Nous avons défini les dépenses de santé catastrophiques comme les paiements directs égaux ou supérieurs à 10% des dépenses des ménages. Les facteurs associés à des dépenses catastrophiques ont été déterminés par analyse multivariable.

**Résultats:**

Globalement, la proportion de dépenses de santé catastrophiques a été multipliée par 1,47 entre l'enquête sur les dépenses de 1993–1994 (12,4%) et celle de 2011–2012 (18,2%), et par 2,24 entre l'enquête sur le recours aux soins de 1995–1996 (11,1%) et celle de même type de 2014 (24,9%). Cette proportion a davantage augmenté dans le quintile le plus pauvre que dans le plus riche, puisqu’elle a été multipliée respectivement par 3,00 et par 1,74, selon les enquêtes sur le recours aux soins. Les dépenses catastrophiques étaient plus courantes dans les foyers composés uniquement de personnes de 60 ans ou plus: le rapport des cotes ajusté (RCa) était de 3,26 (intervalle de confiance de 95%, IC: 2,76–3,84) par rapport aux foyers sans personnes âgées ou avec des enfants de moins de 5 ans. Le risque était également plus élevé pour les foyers composés de personnes âgées et d'enfants (RCa: 2,58; IC 95%: 2,31–2,89), pour ceux qui avaient une femme comme chef de famille (RCa: 1,32; IC 95%: 1,19–1,47) et pour ceux vivant en zone rurale (RCa: 1,27; IC 95%: 1,20-1,35).

**Conclusion:**

En Inde, la proportion de ménages faisant face à des dépenses de santé catastrophiques a augmenté au cours des deux dernières décennies. Ces dépenses étaient plus élevées pour les ménages qui comprenaient des personnes âgées. Il est nécessaire de mettre en place des mécanismes de protection financière pour les groupes de population qui courent un risque d'être confrontés à des dépenses de santé catastrophiques.

## Resumen

**Objetivo:**

Investigar las tendencias en los pagos directos en los servicios sanitarios y el gasto sanitario catastrófico en la India según la composición por edad en el hogar.

**Métodos:**

Se obtuvieron datos de cuatro encuestas nacionales sobre los gastos de los consumidores y tres encuestas de uso de los servicios sanitarios realizadas entre 1993 y 2014. Los hogares se dividieron en cinco grupos según la composición por edad. Se definió el gasto sanitario catastrófico como los pagos directos que igualan o superan el 10% del gasto doméstico. Los factores asociados con el gasto catastrófico se identificaron mediante un análisis multivariable.

**Resultados:**

En general, la proporción del gasto sanitario catastrófico aumentó 1,47 veces entre la encuesta de gastos de 1993-1994 (12,4%) y la encuesta de gastos de 2011-2012 (18,2%) y 2,24 veces entre la encuesta de uso de 1995-1996 (11,1%) y la encuesta de uso de 2014 (24.9%). La proporción aumentó más en el sector más pobre que el más rico: 3,00 veces frente a 1,74 veces, respectivamente, en todas las encuestas de uso. El gasto catastrófico fue más común en los hogares en los que solo había personas de 60 años o más: el coeficiente de posibilidades ajustado, (CPa) fue de 3,26 (intervalo de confidencia (IC) del 95%: 2,76–3,84) en comparación con los hogares sin personas mayores o niños menores de 5 años. El riesgo también aumentó en los hogares con personas mayores y niños (CPa: 2,58; IC del 95%: 2,31–2,89), con una mujer como cabeza de familia (CPa: 1,32; IC del 95%: 1,19-1,47), y en una zona rural (CPa: 1,27; IC del 95%: 1,20–1,35).

**Conclusión:**

La proporción de hogares con un gasto sanitario catastrófico en la India aumentó en las últimas dos décadas. Tal gasto fue mayor en los hogares con personas mayores. Los mecanismos de protección financiera son necesarios en los grupos de población en riesgo de gasto sanitario catastrófico.

## ملخص

**الغرض:**

التحقيق في الاتجاهات السائدة في تكاليف الرعاية الصحية التي يتحملها المريض والنفقات الصحية الجائرة بالهند من خلال التركيبة العمرية لأفراد الأسر.

**الطريقة:**

لقد حصلنا على بيانات من أربعة مسوح وطنية تتعلق بنفقات المستهلكين وثلاثة مسوح تتعلق بمعدل الاستخدام تم إجراؤها في الفترة من عام 1993 إلى عام 2014. وقد تم تقسيم أفراد الأسر إلى خمس مجموعات بحسب التركيبة العمرية لهم. وقمنا بتحديد النفقات الصحية الجائرة بأنها تكاليف يتحملها المريض بنفسه تساوي أو تتجاوز 10‏% من نفقات الأسرة. وتم تحديد العوامل المرتبطة بالنفقات الجائرة من خلال تحليل متعدد المتغيرات.

**النتائج:**

يمكن القول بشكلٍ عام أن نسبة النفقات الصحية الجائرة زادت أضعاف ذلك بمعدل 1.47 في مسح النفقات الذي أجري في الفترة ما بين عام 1993 إلى عام 1994 (بنسبة 12.4‏%) ومسح النفقات الذي أجري في الفترة ما بين عام 2011 إلى عام 2012 (بنسبة 18.2‏%) وأضعاف ذلك بمعدل 2.24 في المسح المتعلق بمعدل الاستخدام الذي أجري في الفترة ما بين عام 1995 إلى عام 1996 (بنسبة 11.1‏%) والمسح المتعلق بمعدل الاستخدام الذي أجري في عام 2014 (بنسبة 24.9‏%). وزادت النسبة أكثر في الفئات الخمسية السكانية الأفقر عن الفئات الخمسية السكانية الأغنى: حيث زادت بمقدار 3 أضعاف بالمقارنة مع الزيادة بواقع 1.74، مرة على الترتيب، وذلك عبر المسوح المتعلقة بمعدل الاستخدام. وكان تكبد النفقات الصحية الجائرة هو الأكثر شيوعًا بين الأسر التي لا تضم سوى أفراد تبدأ أعمارهم من 60 عامًا فما فوق: وكانت نسبة الاحتمالات المعُدلة (aOR) 3.26 (بنسبة أرجحية مقدارها 95‏%، بفاصل ثقة يبلغ: 2.76–3.84) مقارنة بالأسر التي لا تشتمل على كبار السن أو الأطفال الذين تقل أعمارهم عن 5 أعوام. كما زادت الخطورة بين الأسر التي تشتمل على كبار السن والأطفال على حدٍ سواء (نسبة الاحتمالات المعُدلة: 2.58؛ ‏بنسبة أرجحية مقدارها 95‏%: 2.31–2.89)، مع تقدم نسبة الإناث (نسبة الاحتمالات المعُدلة: 1.32؛ ‏بنسبة أرجحية مقدارها 95‏%: 1.19–1.47) ومع التواجد بموقع ريفي (نسبة الاحتمالات المعُدلة: 1.27؛ ‏بنسبة أرجحية مقدارها 95‏%: 1.20–1.35).

**الاستنتاج:**

لقد زادت نسبة الأسر التي تتكبد نفقات صحية جائرة في الهند خلال العقدين الماضيين. وكانت هذه النفقات هي الأعلى بين الأسر التي تشتمل على كبار السن. وينبغي إيجاد آليات مالية لحماية الفئات السكانية المعرضة لخطر تكبد النفقات الصحية الجائرة. ****

## 摘要

**目的:**

旨在按年龄结构调查印度家庭的自费医疗保健支出和灾难性卫生支出的趋势。

**方法:**

我们从 1993 年至 2014 年期间进行的四次全国消费者支出调查和三次卫生保健利用率调查中获得了数据。按年龄结构将所有家庭分为五组。我们将灾难性卫生支出定义为自费支出相当于家庭支出的 10% 或超过 10%。通过多变量分析确定了与灾难性支出相关的因素。

**结果:**

总体而言，1993-1994 年 (12.4%) 和 2011-2012 年支出调查 (18.2%) 之间增加了 1.47 倍，1995–1996 年 (11.1%) 和 2014 年利用率调查 (24.9%) 之间增加了 2.24 倍。最贫穷人群比最富有的五分之一人口的比例增加了：在利用率调查中，这两个数据分别为 3.00 倍和 1.74 倍。与无老年人或年龄小于 5 岁的儿童家庭相比，年龄在 60 岁或以上的家庭中，灾难性支出是最常见的：调整后的优势率 (aOR) 为 3.26 (95% 置信区间，CI:2.76–3.84)。同时拥有老年人和儿童的家庭风险也在增加 (aOR: 2.58; 95% CI: 2.31–2.89)，女性为主 (aOR: 1.32; 95% CI: 1.19–1.47) 和农村人口 (aOR: 1.27; 95% CI: 1.20–1.35)。

**结论:**

过去二十年里，印度家庭的灾难性卫生支出的比例有所增加。老年人家庭的支出是最高的。需要为面临灾难性卫生支出风险的人群提供财务保障机制

## Резюме

**Цель:**

Изучить тенденции в расходах собственных средств пациентов на медицинское обслуживание и катастрофически высоких расходах на здравоохранение с учетом возрастного состава семей в Индии.

**Методы:**

Были получены данные из четырех национальных опросов об уровне потребительских расходов и трех опросов относительно использования услуг здравоохранения, проведенных в период между 1993 и 2014 годами. Все семьи были разделены на пять групп с учетом возрастного состава. Катастрофически высокие расходы на здравоохранение определялись как расходы собственных средств пациентов, равные или превышающие 10% от расходов семьи. Факторы, связанные с катастрофически высокими расходами, были определены с помощью многофакторного анализа.

**Результаты:**

В целом доля катастрофически высоких расходов на здравоохранение увеличилась в 1,47 раза при сравнении результатов изучения потребительских расходов в 1993–1994 годах (12,4%) и 2011–2012 годах (18,2%), а также в 2,24 раза при сравнении исследований использования услуг здравоохранения в 1995–1996 годах (11,1%) и 2014 году (24,9%). Более высокий рост этой доли расходов был отмечен в самом бедном квинтиле по сравнению с самым богатым: в 3,00 раза по сравнению с 1,74 раза соответственно во всех обследованиях использования услуг здравоохранения. Катастрофически высокие расходы были наиболее распространены среди семей, в которых проживали только люди в возрасте 60 лет и старше: скорректированное отношение шансов (сОШ) составило 3,26 (95%-й доверительный интервал, ДИ: 2,76–3,84) по сравнению с семьями без пожилых людей или детей моложе 5 лет. Риск был также выше среди семейств как с пожилыми людьми, так и с детьми (сОШ: 2,58; 95%-й ДИ: 2,31–2,89), с женщиной во главе семьи (сОШ: 1,32; 95%-й ДИ: 1,19–1,47) и в сельской местности (сОШ: 1,27; 95%-й ДИ: 1,20–1,35).

**Вывод:**

В Индии за последние два десятилетия увеличилась доля семей, несущих катастрофически высокие расходы на здравоохранение. Такие расходы были наиболее высокими среди семей с пожилыми людьми. Для групп населения, подверженных риску катастрофически высоких расходов на здравоохранение, требуется создание механизмов финансовой защиты.

## Introduction

Financial catastrophe, or severe financial hardship, can occur in all countries at all income levels. However, its effect is greatest in low-income countries and is more severe in middle- than high-income settings. There is a negative correlation between the proportion of people experiencing financial catastrophe and the extent to which countries fund their health systems by some form of prepayment, such as taxes or insurance.^1^ Accordingly, catastrophic payments are more common in low-income countries where health care is mainly financed by direct payments and less common in high-income countries with established prepayment methods.^2^ In many low- and middle-income countries, a large proportion of health expenditure is paid out of pocket by households. Excessive reliance on out-of-pocket payments can lead to financial barriers for the less well off, thereby increasing inequalities in access to health care, or can result in financial catastrophe or impoverishment.^1^^,^^3^ Estimates from household surveys show that, worldwide each year, around 100 million individuals are impoverished and another 150 million face severe financial difficulties due to direct health expenditure and that more than 90% of people affected live in low-income countries.^1^

Financing health care through out-of-pocket payments results in catastrophic health expenditure and impoverishment in many Asian countries, particularly India.^2^^,^^4^ Out-of-pocket payments remain common in India, where, according to a recent survey, only 15% (50 234/333 104) of the population is covered by health insurance.^5^ In 2014, such payments were estimated to account for 62% of total health expenditure (60.6 billion United States dollars, US$, out of US$ 97.1 billion).^6^ In fact, public expenditure on health in India has remained stagnant at 1% of gross domestic product, far below other emerging BRICS (Brazil, the Russian Federation, India, China and South Africa) economies and lower even than in the neighbouring countries of Nepal and Sri Lanka.^6^

Recent evidence suggests that the changing age distribution of the Indian population is having a substantial effect on health spending.^7^ Identifying population groups at risk of catastrophic health expenditure is important for targeting interventions involving health insurance or other prepayment mechanisms that will counteract the adverse consequences of high out-of-pocket payments. Here we report on recent trends in out-of-pocket payments and catastrophic health expenditure in India using data from nationwide household surveys conducted between 1993 and 2014, with particular reference to household age composition and the identification of households most likely to experience catastrophic health expenditure.

## Methods

We used data from seven national sample surveys, which have been carried out in all Indian states since 1993: four consumer expenditure surveys (referred to as expenditure surveys) and three health-care utilization surveys (referred to as utilization surveys).

### 

#### Consumer expenditure surveys

The four surveys were conducted between 1993 and 1994, between 1999 and 2000, between 2004 and 2005 and between 2011 and 2012, respectively.^8^^–^^11^ We did not use data from the 2009–2010 expenditure surveys because the period was considered an abnormal year for calculating price indices and national income estimates and the survey was therefore repeated in 2011 to 2012.^11^ Each expenditure survey collected data on household expenditure on goods and services for both inpatient and outpatient care. They did not collect information on insurance reimbursements. However, as only 1.3% of households were reported to have had medical expenditure reimbursed in the 2014 utilization survey, household expenditure on health care in the expenditure surveys can be considered as a reasonable approximation of out-of-pocket payments. In the surveys, the recall periods for expenditure on inpatient care were 1 month and 1 year; for outpatient care, the period was 1 month. We used the 1-year recall period for our analysis of expenditure on inpatient care. Details of the items used in the expenditure surveys to assess out-of-pocket payments for inpatient and outpatient care available from the corresponding author. In addition, the surveys collected information on food and non-food items to estimate total household consumption expenditure.

#### Health-care utilization surveys

The three surveys were conducted between 1995 and 1996, in 2004 and in 2014, respectively.^5^^,^^12^^,^^13^ The surveys collected information on the direct expenditure of all individuals in a household pertaining to each episode of hospitalization in a reference period of 1 year and to each outpatient visit for individual ailments in a reference period of 15 days. Out-of-pocket payments on inpatient and outpatient care were obtained after the deduction of any payments reimbursed later. Details of the items used in the utilization surveys to assess out-of-pocket payments for inpatient and outpatient care are available from the corresponding author. In these surveys, only aggregated data on household consumption expenditure were available.

### Variables

Our outcome variables were: per capita out-of-pocket payments for health care in the most recent month; and the occurrence of catastrophic health expenditure in the most recent month. Costs in Indian rupees were expressed in 2014 prices using gross domestic product deflators and then converted into US$ using the average 2014 exchange rate (i.e. 1 US$ = 63.3 Indian rupees).^14^^,^^15^ As inpatient and outpatient expenditure were collected for different recall periods, we converted them into the same recall period of 1 month to calculate per capita out-of-pocket payments and determine whether catastrophic health expenditure had occurred.

In the literature, catastrophic health expenditure is derived in two ways:^2^^,^^16^^–^^23^ out-of-pocket payments are expressed as a proportion either of total household expenditure or of the household’s capacity to pay. Although there is no consensus on the cut-off values for these two proportions, 10% of total household expenditure and 40% of the household’s capacity to pay have been most widely used in previous studies.^2^^,^^17^^,^^18^^,^^20^ We defined catastrophic health expenditure as out-of-pocket payments on health equalling or exceeding 10% of total household expenditure. For a more complete perspective on catastrophic health expenditure, we repeated the analysis based on the household’s capacity to pay (available from the corresponding author).

For the analysis, households were divided into five groups: (i) those with no children (i.e. individuals younger than 5 years) or older people (i.e. individuals aged 60 years or older); (ii) those with children but no older people; (iii) those with older people but no children; (iv) those with both children and older people; and (v) those with older people only. In examining the association between catastrophic health expenditure and household age composition, we took into account several socioeconomic and demographic variables: the age, sex, marital status and educational level of the head of the household, social group (i.e. caste), place of residence, monthly per capita consumption expenditure quintile, the household’s occupation and the type of survey. Monthly per capita consumption expenditure adjusted for household size and composition was used as a proxy for economic status. We used an adjustment factor *e_h_*, where *e_h_* = (*A_h_* + 0.5 *K_h_*)^0.75^, *A_h_* is the number of adults in the household and *K_h_* is the number of children aged 14 years and younger. The parameters 0.5 and 0.75 used in the formula were based on estimates from Deaton.^24^ The 29 Indian states and seven union territories were classified as either less or more developed: the 18 less-developed states included the eight empowered action group states (i.e. Bihar, Chhattisgarh, Jharkhand, Madhya Pradesh, Odisha, Rajasthan, Uttar Pradesh and Uttaranchal), the eight north-eastern states (Arunachal Pradesh, Assam, Manipur, Meghalaya, Mizoram, Nagaland, Sikkim and Tripura) plus Himachal Pradesh and Jammu and Kashmir.^25^

### Statistical analysis

The variation in mean per capita out-of-pocket payments by household age composition and the unadjusted association between catastrophic health expenditure and independent variables derived from survey data are presented as descriptive statistics. We used multivariable logistic regression analysis to investigate the association between catastrophic health expenditure and household age composition after adjustment for other sociodemographic and economic variables in the two most recent surveys: the 2011–2012 expenditure survey and the 2014 utilization surveys. Results are reported as adjusted odds ratios with 95% confidence intervals (CIs). Weighting was applied to the survey data in all analyses to adjust for differences between the composition of the sample and the population surveyed, thereby making estimates representative of the relevant population. The analyses were performed using Stata version 13.1 (StataCorp LP., College Station, United States of America). As our study was based on secondary data from national sample surveys and survey participants could not be identified, exemption from ethics approval was granted by the institutional ethics committees of the Public Health Foundation of India and the London School of Hygiene and Tropical Medicine.

## Results

Households with no children or older people were the most common type: they accounted for 44.3% to 52.6% of households across the seven surveys (Table 1 and Table 2). Households with older people only were least common, accounting for 2.2% to 3.6% of households. Overall, mean per capita out-of-pocket payments by households with older people only were higher than those of all other households: 2.44- to 5.34-fold higher across all utilization surveys and 2.20- to 4.47-fold higher across all expenditure surveys. Mean monthly per capita out-of-pocket payments increased from the poorest to the richest monthly per capita consumption expenditure quintile and the difference in mean payments between the poorest and richest quintiles was highest for households with older people only. In the 2014 utilization survey, mean per capita out-of-pocket payments were 3.95-fold higher in the richest versus the poorest quintile among households with older people only, compared with 2.24-fold higher in households with no children or older people, 3.24-fold higher in households with children but no older people, 2.47-fold higher in households with older people but no children and 3.45-fold higher in households with both children and older people.

**Table 1:** Out-of-pocket health-care payments, by household expenditure and age composition, health-care utilization surveys, India, 1995–2014

Variable	Household composition				
	Households with no children or older people^a^	Households with children but no older people^a^	Households with older people but no children^a^	Households with both children and older people^a^	Households with older people only^a^
**1995–1996 survey (*n* = 120 942)**					
No. of households (%)^b^	50 917 (48.0)	42 564 (30.1)	13 125 (11.4)	12 305 (8.3)	2031 (2.2)
Households with OOP, % (95% CI)^b^	16.7 (16.2–17.3)	21.5 (20.9–22.2)	29.6 (28.2–30.9)	33.9 (32.3–35.5)	22.1 (19.4–24.8)
OOP, mean US$ (SD)^c^					
Poorest quintile	2.6 (3.9)	1.8 (4.0)	2.6 (6.6)	2.5 (8.8)	6.0 (7.1)
Poor quintile	2.9 (10.2)	2.3 (4.7)	2.6 (5.7)	2.1 (3.8)	6.7 (12.2)
Middle quintile	3.7 (11.9)	2.9 (7.6)	3.7 (9.5)	3.1 (12.4)	8.3 (8.8)
Rich quintile	4.1 (15.3)	3.1 (6.9)	3.8 (8.0)	3.1 (6.8)	14.7 (16.1)
Richest quintile	7.7 (19.2)	5.3 (12.3)	7.4 (18.3)	5.2 (9.5)	26.5 (45.6)
All households	4.8 (14.9)	3.2 (7.9)	4.5 (11.9)	3.3 (8.8)	11.7 (22.9)
**2004 survey (*n* = 73 868)**					
No. of households (%)^b^	25 340 (44.3)	20 654 (28.8)	16 990 (15.1)	7 991 (8.7)	2 893 (3.2)
Households with OOP, % (95% CI)^b^	26.9 (26.1–27.7)	51.1 (50.0–52.1)	42.2 (41.1–43.2)	64.5 (62.9–66.2)	31.2 (29.0–33.4)
OOP, mean US$ (SD)^c^					
Poorest quintile	2.9 (5.5)	1.7 (3.5)	3.4 (6.1)	2.0 (4.1)	7.1 (10.7)
Poor quintile	3.8 (7.4)	2.0 (3.8)	4.4 (10.3)	2.1 (3.4)	9.2 (12.0)
Middle quintile	4.0 (7.6)	3.4 (14.9)	4.4 (7.2)	2.9 (4.6)	9.8 (12.9)
Rich quintile	5.5 (10.9)	3.3 (6.2)	5.6 (9.9)	3.3 (5.2)	17.1 (31.4)
Richest quintile	8.0 (19.2)	4.4 (8.8)	9.6 (19.9)	5.3 (8.7)	31.9 (73.6)
All households	5.2 (12.4)	2.9 (8.8)	6.0 (13.1)	3.3 (5.8)	15.5 (41.0)
**2014 survey (*n* = 65 932)**					
No. of households (%)^b^	24 139 (50.7)	20 930 (22.0)	10 648 (16.5)	8 536 (7.4)	1 679 (3.4)
Households with OOP, % (95% CI)^b^	31.7 (30.6–32.8)	53.1 (51.4–54.9)	52.5 (50.3–54.6)	66.9 (63.9–69.9)	49.7 (45.1–54.3)
OOP, mean US$ (SD)^c^					
Poorest quintile	5.4 (15.9)	2.9 (5.3)	6.0 (14.3)	3.3 (7.8)	9.7 (15.1)
Poor quintile	4.7 (9.7)	3.7 (6.3)	5.0 (7.6)	5.1 (9.7)	11.6 (25.8)
Middle quintile	5.7 (10.1)	4.0 (6.5)	6.8 (20.2)	4.5 (6.8)	21.8 (46.7)
Rich quintile	6.6 (12.7)	5.6 (11.6)	7.7 (14.3)	5.5 (9.0)	21.4 (27.6)
Richest quintile	12.1 (27.7)	9.4 (15.9)	14.8 (31.6)	11.4 (18.3)	38.3 (50.5)
All households	7.0 (17.0)	4.7 (9.4)	8.7 (20.9)	5.7 (11.0)	21.6 (38.3)

CI: confidence interval; OOP: out-of-pocket payments; SD: standard deviation; US$: United States dollar.

^a^ Children were individuals younger than 5 years and older people were individuals aged 60 years or older.

^b^ The percentages shown are weighted percentages, which make the estimates representative of the relevant population.

^c^ Per capita out-of-pocket payments in the month before the survey and values are only for households that made out-of-pocket payments.

**Table 2:** Out-of-pocket health-care payments, by household expenditure and age composition, consumer expenditure surveys, India, 1993–2012

Variable	Household composition				
	Households with no children or older people^a^	Households with children but no older people^a^	Households with older people but no children^a^	Households with both children and older people^a^	Households with older people only^a^
**1993–1994 survey (*n* = 115 354)**					
No. of households (%)^b^	52 678 (44.4)	32 768 (30.2)	16 109 (13.3)	11 255 (9.6)	2 544 (2.5)
Households with OOP, % (95% CI)^b^	52.3 (51.7–52.9)	64.2 (63.5–64.8)	71.3 (70.2–72.4)	63.4 (62.4–64.3)	51.8 (49.5–54.2)
OOP, mean US$ (SD)^c^					
Poorest quintile	0.5 (0.6)	0.4 (0.5)	0.6 (0.7)	0.4 (0.4)	1.2 (1.1)
Poor quintile	0.8 (0.8)	0.6 (0.7)	0.7 (0.8)	0.6 (0.6)	2.1 (2.0)
Middle quintile	1.0 (1.1)	0.9 (1.0)	1.0 (1.1)	0.8 (0.8)	2.5 (2.5)
Rich quintile	1.4 (1.6)	1.3 (1.4)	1.4 (1.5)	1.2 (1.3)	3.7 (3.5)
Richest quintile	2.8 (5.4)	2.7 (4.7)	2.9 (4.4)	2.3 (3.3)	8.9 (15.4)
All households	1.5 (3.2)	1.2 (2.3)	1.5 (2.6)	1.1 (1.8)	3.3 (7.1)
**1999–2000 survey (*n* = 120 307)**					
No. of households (%)^b^	56 933 (46.2)	30 324 (27.1)	18 407 (14.5)	11 749 (9.5)	2 894 (2.8)
Households with OOP, % (95% CI)^b^	63.0 (62.4–63.6)	74.6 (74.0–75.3)	74.3 (73.4–75.2)	82.0 (80.9–83.0)	67.8 (65.6–70.0)
OOP, mean US$ (SD)^c^					
Poorest quintile	0.4 (0.6)	0.4 (0.5)	0.5 (0.6)	0.4 (0.5)	1.1 (1.1)
Poor quintile	0.7 (0.8)	0.6 (0.7)	0.7 (0.8)	0.6 (0.7)	1.8 (1.8)
Middle quintile	0.9 (1.1)	0.9 (1.0)	1.0 (1.2)	0.8 (0.9)	2.6 (2.6)
Rich quintile	1.4 (1.8)	1.2 (1.5)	1.4 (1.8)	1.1 (1.4)	4.4 (4.7)
Richest quintile	2.8 (9.2)	2.4 (4.9)	2.8 (5.3)	2.1 (4.1)	8.6 (24.5)
All households	1.4 (4.8)	1.1 (2.3)	1.4 (3.0)	1.1 (2.2)	3.3 (10.9)
**2004–2005 survey (*n* = 124 644) **					
No. of households (%)^b^	60 568 (48.4)	29 561 (24.9)	19 512 (14.9)	11 437 (8.7)	3 566 (3.1)
Households with OOP, % (95% CI)^b^	65.8 (65.2–66.4)	67.1 (66.3–67.9)	66.5 (65.5–67.5)	68.9 (67.7–70.2)	65.8 (63.5–68.1)
OOP, mean US$ (SD)^c^					
Poorest quintile	0.7 (0.8)	0.6 (0.7)	0.6 (0.8)	0.5 (0.6)	1.1 (1.1)
Poor quintile	1.0 (1.2)	0.9 (1.1)	1.1 (1.3)	0.9 (1.1)	1.4 (1.3)
Middle quintile	1.5 (1.8)	1.4 (1.7)	1.6 (2.0)	1.4 (2.0)	2.3 (2.8)
Rich quintile	2.4 (3.0)	2.1 (2.7)	2.3 (2.9)	2.2 (2.8)	3.0 (3.2)
Richest quintile	5.9 (13.4)	4.7 (8.6)	6.8 (16.9)	4.4 (9.3)	7.5 (12.0)
All households	2.3 (6.4)	1.6 (3.5)	2.0 (6.5)	1.2 (2.8)	5.3 (9.5)
**2011–2012 survey (*n* = 101 662)**					
No. of households (%)^b^	53 365 (52.6)	19 100 (20.2)	18 209 (16.8)	7 922 (6.9)	3 066 (3.6)
Households with OOP, % (95% CI)^b^	75.1 (74.3–75.8)	86.4 (85.5–87.3)	86.4 (85.5–87.4)	91.7 (90.6–92.8)	83.4 (81.0–85.8)
OOP, mean US$ (SD)^c^					
Poorest quintile	0.7 (0.8)	0.7 (0.7)	0.8 (0.9)	0.7 (0.7)	2.1 (1.9)
Poor quintile	1.0 (1.2)	1.0 (1.1)	1.2 (1.4)	1.1 (1.3)	3.6 (3.0)
Middle quintile	1.6 (2.1)	1.5 (1.8)	1.8 (2.3)	1.7 (1.9)	5.3 (4.8)
Rich quintile	2.4 (3.2)	2.4 (2.8)	3.0 (3.9)	2.7 (3.1)	9.0 (7.9)
Richest quintile	6.0 (13.6)	5.5 (9.5)	8.2 (17.8)	7.1 (10.4)	24.1 (33.9)
All households	2.4 (6.8)	1.9 (4.1)	3.0 (8.7)	2.6 (5.2)	8.5 (18.1)

CI: confidence interval; OOP: out-of-pocket payments; SD: standard deviation; US$: United States dollar.

^a^ Children were individuals younger than 5 years and older people were individuals aged 60 years or older.

^b^ The percentages shown are weighted percentages, which make the estimates representative of the relevant population.

^c^ Per capita out-of-pocket payments in the month before the survey and values are only for households that made out-of-pocket payments.

**Fig. 1:**
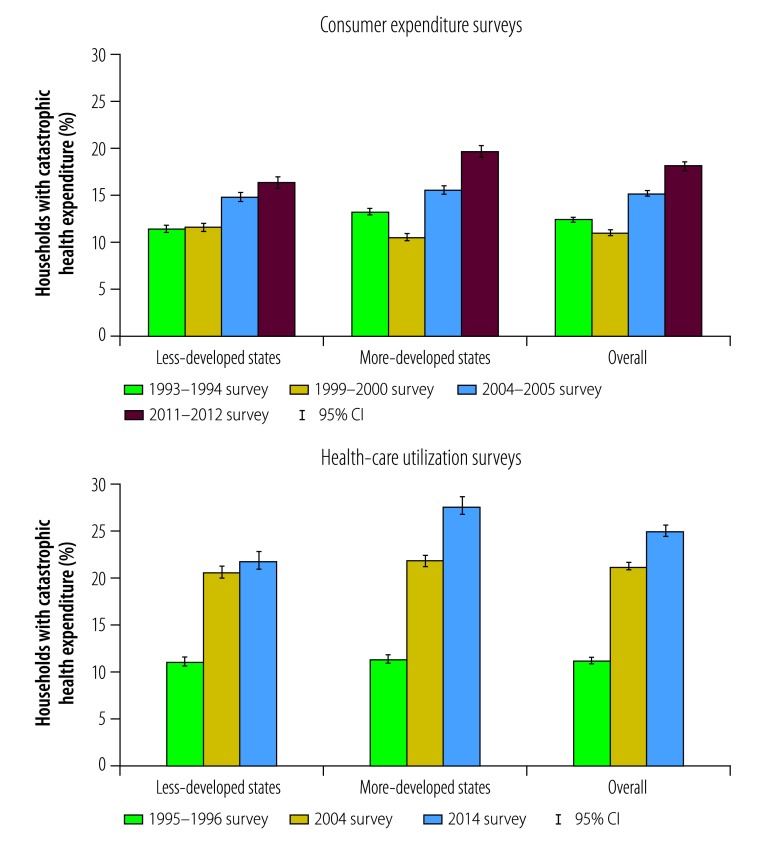
Proportion of households with catastrophic health expenditure, by state’s level of development, India, 1993–2014

Overall, the proportion of households with catastrophic health expenditure increased 1.47-fold between the 1993–1994 expenditure survey and the 2011–2012 expenditure survey and 2.24-fold between the 1995–1996 utilization survey and the 2014 utilization survey (Fig. 1). The proportion increased more between the 1995–1996 and the 2004 utilization survey than between the 2004 and the 2014 utilization survey: 1.91-fold versus 1.17-fold, respectively. The proportion of catastrophic health expenditure was 1.39-fold higher in the 2004 utilization survey than the 2004–2005 expenditure survey. In addition, the increase in proportion between the 1995–1996 and the 2014 utilization survey was greater in more-developed than less-developed states (2.45-fold versus 1.98-fold, respectively), which increased the difference between them. In the 1995–1996 utilization survey, the proportion of households experiencing catastrophic health expenditure was similar in the two groups of states but, in the 2014 utilization survey, it was 1.27-fold higher in more-developed states.

CI: confidence interval.

Notes: Percentages are weighted. Catastrophic health expenditure was defined as out-of-pocket payments on health in the recall period of 1 month equalling or exceeding 10% of total household expenditure. The 29 Indian states and seven union territories were classified as either less or more developed: the 18 less-developed states included the eight empowered action group states (i.e. Bihar, Chhattisgarh, Jharkhand, Madhya Pradesh, Odisha, Rajasthan, Uttar Pradesh and Uttaranchal), the eight north-eastern states (Arunachal Pradesh, Assam, Manipur, Meghalaya, Mizoram, Nagaland, Sikkim and Tripura) plus Himachal Pradesh and Jammu and Kashmir.^25^

**Fig. 2:**
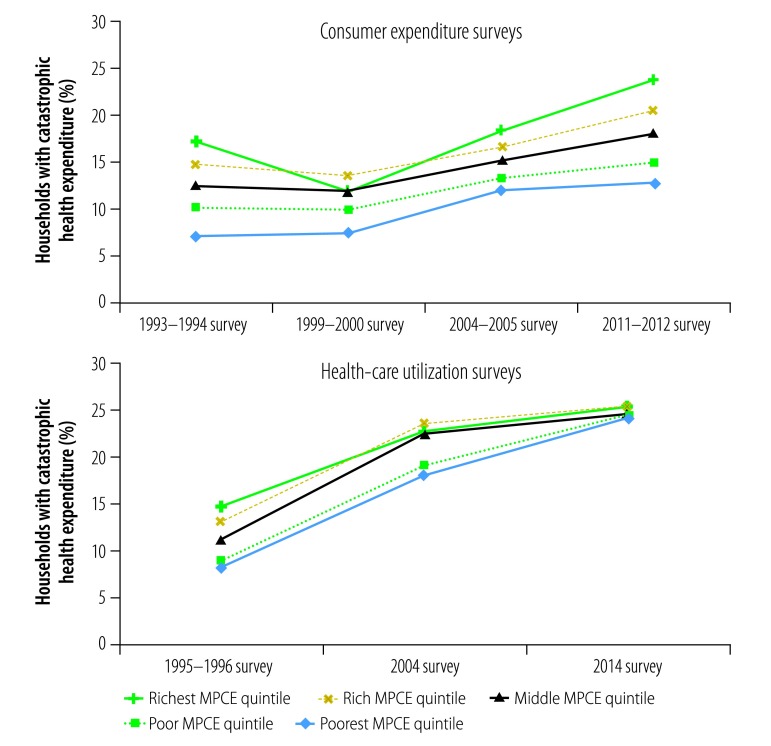
Proportion of households with catastrophic health expenditure, by monthly per capita consumption expenditure quintile, India, 1993–2014

Generally, catastrophic health expenditure was more frequent among households in the richest quintile than among those in the poorest in all expenditure surveys (range: 1.54- to 2.45-fold more frequent) and all utilization surveys (range: 1.03- to 1.78-fold more frequent; Fig. 2). However, the gap decreased over time because the proportion of households experiencing catastrophic health expenditure increased more in the poorest than the richest quintile. Between the 1993–1994 and the 2011–2012 expenditure survey, the proportion increased 1.84-fold in the poorest quintile compared with 1.38-fold in the richest. Between the 1995–1996 and the 2014 utilization survey, the proportion increased 3.00-fold in the poorest quintile and 1.74-fold in the richest.

MPCE: monthly per capita consumption expenditure.

Notes: Percentages are weighted. Catastrophic health expenditure was defined as out-of-pocket payments on health in the recall period of 1 month equalling or exceeding 10% of total household expenditure.

Multivariable analysis showed that, after adjusting for other covariates, the odds of catastrophic health expenditure in a household with older people only compared to a household with no children or older people was 3.26 (95% CI: 2.76–3.84; Table 3) and the odds in a household with both children and older people was 2.58 (95% CI: 2.31–2.89). In addition, richer households were significantly more likely to incur catastrophic health expenditure, as were households that were headed by females, had members who were casual labourers or were in rural areas or in more-developed states. The adjusted odds of catastrophic health expenditure in the 2014 utilization survey compared with the 2011–2012 expenditure survey was 1.54 (95% CI: 1.46–1.62).

**Table 3:** Association between catastrophic health expenditure and demographic and socioeconomic variables, by multivariable analysis, India, 2011–2014

Variable	No. of households (%)^a^ (*n* = 167 594)	No. of households with catastrophic health expenditure (%)^a,b,c^	Risk of catastrophic health expenditure, aOR (95% CI)
**Survey**			
2011–2012 CES	101 662 (50.2)	16 838 (18.2)	Reference
2014 HUS	65 932 (49.8)	31 628 (24.9)	1.54 (1.46–1.62)
**Household age composition**			
No children or older people^d^	77 504 (51.6)	16 116 (15.5)	Reference
With children but no older people	40 030 (21.1)	13 201 (23.8)	1.76 (1.65–1.88)
With older people but no children	28 857 (16.6)	9 938 (27.7)	1.93 (1.76–2.12)
With both children and older people	16 458 (7.2)	6 853 (33.9)	2.58 (2.31–2.89)
Older people only	4 745 (3.5)	2 358 (41.7)	3.26 (2.76–3.84)
**Place of residence**			
Urban	71 419 (31.9)	20 810 (20.4)	Reference
Rural	96 175 (68.1)	27 656 (22.0)	1.27 (1.20–1.35)
**Sex of head of household**			
Male	148 315 (88.0)	42 212 (21.0)	Reference
Female	19 279 (12.0)	6 254 (25.0)	1.32 (1.19–1.47)
**Age of head of household**			
< 60 years	133 488 (81.5)	34 910 (19.0)	Reference
≥ 60 years	34 106 (18.5)	13 556 (32.7)	1.14 (1.04–1.26)
**Marital status of head of household**^e^			
Other	24 884 (15.8)	7 339 (21.3)	Reference
Currently married	142 708 (84.2)	41 127 (21.5)	1.34 (1.22–1.47)
**Caste of household**^e,f^			
Scheduled caste or tribe	48 766 (27.9)	12 000 (19.2)	Reference
Not scheduled caste or tribe	118 814 (72.1)	36 465 (22.4)	1.14 (1.07–1.21)
**Education of head of household**^e^			
Literate	118 788 (66.4)	32 127 (20.9)	Reference
Illiterate	41 707 (33.6)	12 953 (22.6)	1.07 (1.01–1.14)
**Household's occupation**^e^			
Regular wage or salary	42 795 (19.5)	11 075 (19.4)	Reference
Self-employed	79 345 (46.2)	22 990 (21.5)	1.04 (0.97–1.12)
Casual labour	33 287 (26.9)	9 914 (21.0)	1.17 (1.07–1.27)
Other	12 140 (7.4)	4 482 (29.1)	1.22 (1.09–1.37)
**Wealth quintile**^e,g^			
Poorest	24 813 (20.2)	6 639 (18.8)	Reference
Poor	28 871 (19.9)	7 824 (19.7)	1.09 (1.00–1.19)
Middle	33 274 (20.0)	9 093 (21.5)	1.27 (1.17–1.39)
Rich	37 957 (20.0)	11 051 (23.0)	1.44 (1.32–1.57)
Richest	42 669 (20.0)	13 859 (24.6)	1.82 (1.66–2.00)
**State’s level of development**			
Less developed	86 652 (46.0)	21 359 (19.1)	Reference
More developed	80 942 (54.0)	27 107 (23.6)	1.28 (1.21–1.35)

CES: consumer expenditure survey; CI: confidence interval; HUS: health-care utilization survey; OOP: out-of-pocket payments; aOR: adjusted odds ratio.

^a^ The percentages shown are weighted percentages, which make the estimates representative of the relevant population.

^b^ Catastrophic health expenditure was defined as OOP payments on health in the recall period of 1 month equalling or exceeding 10% of total household expenditure.

^c^ The percentage listed is the percentage of the total number of households in the category.

^d^ Children were individuals younger than 5 years and older people were individuals aged 60 years or more.

^e^ Data were missing on marital status for 2 households, on caste for 14, on the education of the head of the household for 7099, on the household’s occupation for 27 and on monthly per capita consumption expenditure for 10.

^f^ The community was stratified socially into four groups according to caste: scheduled castes, scheduled tribes, other backward castes and other castes. Scheduled castes and tribes are officially designated as disadvantaged groups in India.

^g^ The wealth quintiles were calculated in the following way: household’s total monthly consumption expenditure was adjusted for household size and composition to calculate the per capita household consumption expenditure in a month. This was then divided into quintiles and used as a measure of economic status of the household.

Note: Inconsistencies arise in some values due to rounding.

## Discussion

The provision of universal health coverage depends on measuring and monitoring the catastrophic implications of high out-of-pocket payments for health care. This study provides data on trends in out-of-pocket payments and catastrophic health expenditure in India since 1993 and identifies those households most susceptible to catastrophic expenditure. Three key findings emerge. First, the proportion of households experiencing catastrophic health expenditure increased in the 20 years up to 2014, the increase was greater for the poor than the rich. Second, the proportion was highest among households with older people. Third, the odds of catastrophic health expenditure were also higher in households headed by females and in rural households, both factors relevant to policy.

The Indian government is unable to cover the full spectrum of health-care needs because of persistently low public investment in health, a lack of human resources and poor health infrastructure, which increase the cost and the financial burden of care.^26^ In 2015, an estimated 8% of the Indian population had been pushed below the poverty line by high out-of-pocket payments for health care.^27^ The relatively greater increase in catastrophic health expenditure among the poor that we found is important for policy. Therefore, one can argue that the introduction of nationwide health programmes in India to protect poor and marginalized groups against the high cost of health care, such as the National Rural Health Mission in 2005 and Rashtriya Swasthya Bima Yojana in 2008, have not been very effective. However, in areas where the institutional capacity to organize mandatory nationwide risk-pooling is weak, community-based health insurance schemes can be effective in protecting poor households from unpredictably high medical expenses.^22^ Strengthening the ability of health-care systems to provide comprehensive care by increasing investment and human resources is essential for reducing the burden of catastrophic health expenditure.

The high health-care expenditure we found in households with older people and the resulting increased financial burden are particularly relevant today given India’s ageing demographic profile. A previous study using data from the 1999–2000 expenditure survey also showed that the monthly per capita health spending of households with older people only was 3.8-fold higher than that of households with no older people.^28^ Some argue that older people spend more because old age is associated with deteriorating health and a higher burden of disease and disability.^29^^–^^31^ In contrast, others argue that health expenditure does not rise with age per se, but that people close to death, who are older on average, tend to have greater health expenditure.^32^^–^^34^ In addition, older people are less likely to work if they are unhealthy, which could increase the economic burden on their families and society.^35^ Evidence from low- and middle-income countries indicates that households with older people, especially those with chronic noncommunicable diseases or disabilities, experience higher rates of catastrophic health expenditure.^17^^,^^35^^–^^37^ Even in some of the wealthiest countries in Europe, older people diagnosed with chronic diseases face catastrophic health expenditure.^38^ In coming decades, an ageing population combined with the absence of active measures to reduce catastrophic health expenditure will result in more older people falling into poverty and poor health.^21^

Knowledge of the population at risk of catastrophic health expenditure is important for targeting preventative health interventions and for providing protective financial interventions through prepayment schemes. The decision on whether or not to seek health care usually involves several household members, with the head of the household playing a critical role.^39^ We found that households headed by females were at a higher risk of catastrophic health expenditure, indicating that there are gender differences in the capacity to pay for health care. Moreover, catastrophic health expenditure was more common in rural households, which are often doubly disadvantaged because their health needs are greater but their economic resources are severely constrained.^40^ The higher frequency of catastrophic health expenditure we found in more-developed states may have been due to the availability of more extensive health services with better physical access, which increased utilization. Although increasing the availability of health services in less-developed states is important for improving health-care use, households also need to be protected against the adverse consequences of high out-of-pocket payments.

Some studies report that catastrophic health expenditure is more common among the poor,^41^^–^^45^ whereas others report it being more common among the rich.^2^^,^^17^^,^^46^^,^^47^ We found that the proportion of catastrophic health expenditure increased with monthly per capita consumption expenditure, even after adjustment for other covariates. The higher proportion among the rich illustrates the inequities in access to health care that can arise when payments are made out of pocket.^48^ Better-off households can respond more often to medical needs, but are less likely to face permanent impoverishment. Whereas, without adequate resources, poor households simply choose to forgo health care to avoid catastrophic health expenditure in the short run, which could have severe long-term consequences for health and earnings. The adverse impact of ill health in poorer households is grossly underestimated because it is not included in identifying catastrophic health expenditure.^49^

We found that the proportion of catastrophic health expenditure was higher in the utilization surveys than the expenditure surveys, which suggests that the survey design, choice of recall period and number of items used to derive health expenditure should all be taken into account when out-of-pocket payments and catastrophic health expenditure are compared across different types of survey or between different times for the same survey type.^20^^,^^48^^,^^50^ As reported elsewhere, our study also found that the proportion of catastrophic health expenditure was sensitive to the definition used.^20^^,^^48^ Better understanding of the distribution of catastrophic health expenditure could be obtained by exploring the effect of different definitions and thresholds.

Our study has some limitations. First, as the calculation of out-of-pocket payments did not include indirect costs such as the loss of household income, the proportion of catastrophic health expenditure may have been underestimated. Second, as our estimation of the proportion considered only households that incurred health expenditure, the adverse impact of health-care costs on those who did not seek treatment because they could not afford it was not examined. Third, expenditure data were self-reported and could not be verified from other sources. Fourth, ideally the extent to which living standards are seriously disrupted by expenditure on health care in response to illness shocks should be estimated using longitudinal data. However, in the absence of such data, repeated cross-sectional studies can provide a fairly reliable estimate of trends in catastrophic health expenditure.

Despite these limitations, our study provides evidence that has important policy implications for India as well as for other low- and middle-income countries undergoing the demographic and economic transition. Older people are less able to bear the cost of health care because they lack a stable income and are more economically dependent. Higher public expenditure on health, the provision of affordable health care and an improved geriatric health infrastructure are required. In addition, governments should provide financial protection through viable prepayment mechanisms and risk-pooling and ensure health security for the population younger than 60 years, particularly for children younger than 5 years. To achieve equity in health-care financing, public policy should focus on economically disadvantaged groups. Insurance coverage and the provision of good-quality, subsidized, public health facilities will both improve access to health care and protect the poor against financial catastrophe. These actions are important for improving health in India and for achieving the sustainable development goals set by the United Nations.
